# Importance of an Ongoing Nutritional Counselling Intervention on Eating Habits of Newly Diagnosed Children with Celiac Disease

**DOI:** 10.3390/nu16152418

**Published:** 2024-07-25

**Authors:** Gesala Perez-Junkera, Edurne Simón, Ariane Erika Calvo, Zuriñe García Casales, Pablo Oliver Goicolea, Juan Ignacio Serrano-Vela, Idoia Larretxi, Arrate Lasa

**Affiliations:** 1 GLUTEN3S Research Group, Department of Nutrition and Food Science, University of the Basque Country, 01006 Vitoria-Gasteiz, Spain; gesala.perez@ehu.eus (G.P.-J.); edurne.simon@ehu.eus (E.S.); arrate.lasa@ehu.eus (A.L.); 2 Bioaraba, Nutrición y Seguridad Alimentaria, 01006 Vitoria-Gasteiz, Spain; 3 Children’s National Hospital, 111 Michigan Avenue NW, Washington, DC 20010, USA; 4 Section of Gastroenterology, Hepatology and Nutrition, Pediatric Services, University Hospital of Araba, 01009 Vitoria-Gasteiz, Spain; arianeerika.calvosaez@osakidetza.eus (A.E.C.); zurine.garciacasales@osakidetza.eus (Z.G.C.); 5 Pediatric Services, Hospital of Mendaro-OSI Debabarrena, 20850 Mendaro, Spain; pablo.olivergoicolea@osakidetza.eus; 6 Coeliac Disease and Gluten Sensitivity Association of Madrid, 28028 Madrid, Spain; nachoserrano@celiacosmadrid.org

**Keywords:** celiac disease, gluten-free diet, dietary adherence, follow-up, intervention, nutritional balance, nutrition education

## Abstract

A strict lifelong gluten-free diet (GFD) is the current treatment for the management of celiac disease (CD). Several studies have demonstrated that without proper dietary assessment, this diet leads to nutritional deficiencies and/or imbalances. The present study aimed to improve the dietary habits of newly diagnosed children with CD through ongoing and face-to-face dietary counseling. Forty-three participants were followed during the first year after CD diagnosis. Dietary data were collected at diagnosis (Vt0), after 3 months on a GFD (Vt3), and after 1 year following a GFD (Vt12). Participants completed a 3-day 24-h food recall, a food frequency questionnaire, and the KIDMED index. After each data collection, participants received dietary assessment and nutritional education. Participants consumed more plant-origin foods after the intervention, with most of them reaching the daily recommendations. Fresh food intake increased and that of ultra-processed foods decreased. Compliance with the Mediterranean diet also improved. Personalized dietary assessment and ongoing follow-up improved the dietary patterns of children recently diagnosed with CD, highlighting the importance of dietitian involvement in the management of CD.

## 1. Introduction

Celiac disease (CD) is a condition in which people with a genetic predisposition develop an immune reaction to dietary gluten (a complex storage protein, composed of gliadins and glutenins, found in some cereals). In childhood, clinical presentations vary with age, ranging from intestinal symptoms (abdominal pain, bloating, vomiting, or failure to thrive) to extra-intestinal symptoms such as osteopenia, iron deficiency, anemia, or neurological/psychological disorders [1,2,3]. Symptoms range from severe presentations to asymptomatic individuals diagnosed by screening high-risk groups [4]. Over the last two decades, CD has been a major public health problem, and its incidence is on the rise worldwide. This is not only due to environmental factors together with unhygienic conditions but also because diagnosis techniques have improved [5,6,7]. Prevalence may vary by location, gender, and age. It affects an estimated 1% of Western populations, with a global biopsy or serology detection rate of 0.7–1.4% [8,9,10]. However, with a 1/3 to 1/5 ratio of diagnosed to undiagnosed cases, the disease sometimes goes undetected [11]. In addition, the biopsy-proven prevalence of CD was found to be 1.5 times higher in women than in men and approximately twice as high in children than in adults [10,12].

Currently, the only treatment for the disease is following a lifelong gluten-free diet (GFD). Gluten is found in some cereals. These include wheat, rye, barley, spelt, kamut, and their hybridized varieties [13]. In the case of oats, there is some controversy relating to their inclusion or exclusion from the GFD [14] since, in some regions, it is considered to not be harmful to the majority of patients [15,16,17]. In addition to familiar wheat-based foods such as bread, pasta, bakery products, and other processed snacks, gluten acts as a structure protein and gives dough its viscoelastic properties [18,19]. GFD includes foods that are naturally gluten-free (e.g., gluten-free cereals, pulses, fruit and vegetables, unprocessed meat, fish, eggs, and dairy products) and/or wheat-based alternatives that are specially manufactured to be gluten-free and contain less than 20 ppm of gluten, as required by European legislation [20,21]. In addition, GFD must have two characteristics [22]: it must ensure the total absence of gluten, which is essential for the improvement of the duodenal mucosa and the disappearance of symptoms [23,24], and it must be nutritionally balanced. However, unbalanced percentages of macronutrients and various vitamin and mineral inadequacies have been observed. In particular, studies in children have confirmed that GFD is typically characterized by low carbohydrate, fiber, iron, calcium, folate, niacin, and zinc intakes and excessive saturated fat [25,26]. In addition, in young children, the exclusion of cereals containing gluten, combined with the common consumption of highly processed gluten-free foods of poor nutritional value, can contribute to adverse health effects of GFD [27,28]. In line with this, Larretxi et al. concluded that although the diet of children and adolescents with CD was unhealthy because of inadequate dietary habits, following a diet composed of GF products posed additional difficulties in meeting nutritional guidelines [22]. Therefore, the role of dietitians specializing in digestive diseases will be crucial, not only to establish appropriate dietary guidelines but also to provide reliable dietary education, monitor ongoing treatment [27,29], and help in detecting unintentional gluten ingestions [30]. However, studies where people with CD were given specific dietary guidelines over a period of time are scarce, and those present in the literature did not observe an improvement of the whole dietary profile, suggesting the need for a closer follow-up [31,32].

Taking into account all of the above-mentioned factors, continuous nutritional assessment of individuals living with this condition, especially if it is carried out at an early age, is crucial for promoting healthy eating habits and reaching a better quality of life [33]. Therefore, the aim of the present work was to provide ongoing and face-to-face dietary counseling to newly diagnosed children with CD to improve their dietary habits. Moreover, we wanted to educate them and their families about CD and GFD in order to improve their knowledge and promote self-care.

## 2. Materials and Methods

### 2.1. Participants and Recruitment Centers

People with CD were recruited between 2020 and 2023. Almost all newly diagnosed children from six hospitals from the Basque Country (Hospital Universitario de Cruces, Hospital Universitario de Donostia, Hospital Universitario Araba, Hospital del Alto Deba, Hospital de Mendaro, Hospital de Zumarraga) and children from the Coeliac Disease and Gluten Sensitivity Association Madrid took part in the study. All participants (n = 43; 33 girls and 10 boys) were referred to the clinics for confirmation of a diagnosis of CD during this period (2020–2023). The mean age of participants of participants was 8.2 ± 3.2 years and their distribution according to age was as follows: <5 years: 6 participants (14%), 5–10 years: 23 participants (53%), >10 years: 14 participants (33%).

Inclusion criteria were (i) being a child who volunteered to participate in the intervention study, (ii) being a child with a recent diagnosis of CD, and (iii) being willing to follow a GFD. Exclusion criteria included (i) a history of chronic diseases (e.g., cardiovascular diseases, diabetes, hyperthyroidism/hypothyroidism, hypercholesterolemia, hypertriglyceridemia, or hypertension) or other digestive pathologies requiring specific dietary advice and (ii) lacking a desire to be part of the intervention. Written informed consent was obtained from parents and/or tutors of all participants after they had received information about the study. Minor participants signed an approval of participation. This study was approved by the Ethical Committee of The Basque Country (Comité Ético de Investigación con Medicamentos de Euskadi -CEIC-), Code PI2020053.

### 2.2. Data Collection

After confirmation of a diagnosis of CD and acceptance to participate in the intervention, participants were enrolled on a consecutive basis and followed for the first 12 months of treatment. All participants attended three medical/dietary visits: at diagnosis (Vt0), three months after a GFD (Vt3), and 12 months after the introduction of GFD (Vt12). Anthropometric measurements, energy expenditure, and biochemical data were collected from the participants at the medical visits.

Dietary data were also collected at diagnosis (Vt0), three months after a GFD (Vt3), and 12 months after a GFD (Vt12). Participants were asked to complete specific dietary questionnaires on each occasion and the records were told to be sent within a few days after each visit. Questionnaires were sent by post or email once completed, and specialized dietitians provided a personalized dietary report with nutritional advice.

### 2.3. Intervention Design

The intervention consisted of face-to-face follow-up meetings with the participants of each recruitment center. These meetings were carried out after each round of data collection, one month after diagnosis (first intervention, IT1), one month after the third month on a GFD (second intervention, IT4), and after a year on a GFD (third intervention, IT13)). The intervention aimed to familiarize the participants and their families with CD and the GFD and provide continuous nutritional assessments to improve the participant’s nutritional status and the quality of the diet and promote self-care. Two dietitians specialized in CD and GFD from the GLUTEN3S research team at the UPV/EHU analyzed dietary data and carried out this intervention. Common criteria for dietary data analysis were established before the study, and dietitians co-worked during the whole intervention in order to avoid interpersonal differences.

In the first meeting (IT1), basic concepts of CD, gluten, and the GFD, as well as the guidelines for achieving a balanced diet, were defined. In addition, a didactic workshop on different flours and the comparison of organoleptic characteristics made it possible to highlight the differences between a gluten-free product and a gluten-containing product.

At IT4, the focus was on the safety of the GFD; in particular, topics such as cross-contamination, gluten-free labeling, current legislation, and the possible sources of gluten allergies were discussed. At this second meeting, another didactic and video workshop was held. If there were children whose diet had been identified as “high risk” for involuntary transgressions, they were informed and given the guidelines to follow to minimize this risk.

At IT13, data obtained over the 12 months on a GFD were presented to the participants.

Gluten-free cooking workshops were organized between IT1 and IT4 for all study participants and broadcasted via the Zoom Platform. Specifically, gluten-free recipes were prepared, including a shopping session in the supermarket to understand food labeling and recognize gluten-free foods, the cooking session itself, with an emphasis on cross-contamination, and finally, tasting the prepared food.

At the end of each session, the families were delivered relevant information about the content of each session. Families also had the opportunity to analyze their personal diets with the dietitian and were given advice on how to balance their diets.

### 2.4. Anthropometric Measurements

Trained professionals obtained anthropometric measurements. Body weight (±10 g) was estimated by a digital integrating scale (SECA 760). Height was calculated to the nearest 5 mm by a stadiometer (SECA 220). The Body Mass Index (BMI) was calculated from weight and height (kg/m^2^) and expressed as a percentile according to the classification of Sobradillo et al. [34].

### 2.5. Calculation of Energy Expenditure

In the SEGHNP (Sociedad Española de Gastroenterología, Hepatología y Nutrición Pediátrica) Nutritional Application for use in pediatric consultations [35], the Harris–Benedict equation method was used to estimate the total energy expenditure of the participants, taking into account “intense physical activity”.

### 2.6. Biochemical Data

Fasting glucose, total cholesterol, HDL-cholesterol (HDL-c), LDL-cholesterol (LDL-c), triglycerides, and ferritin levels were measured at each visit. The values were compared with references to the Basque Health System.

### 2.7. Dietary Data

Dietary intake was assessed using 3-day 24-h food recalls (on 2 working days and 1 weekend day), a Food Frequency Questionnaire (FFQ), and the KIDMED questionnaire. So as to avoid bias, dietitians assisted participants in measuring the diet, food portions, and quantities using photographs of rations and sizes described in Rusolillo and Marques´ Photo Album [36]. Dietary Reference Intakes (DRIs) for the Spanish population issued by the Spanish Societies of Nutrition, Feeding, and Dietetics (FESNAD) were used as a reference for the interpretation of the micronutrient intakes [37]. In the case of the FFQ, the recommendations of the Spanish Society of Community Nutrition (SENC) were applied for an accurate interpretation of the results [38].

Moreover, the energy contribution of the GFP (Gluten-free product) was determined for each participant, and the food and drinks consumed were classified based on the NOVA system [39]. NOVA classifies foods on the basis of their nature, purpose, and extent of industrial processing, and it differentiates between the following groups: (i) unprocessed or minimally processed foods, (ii) processed culinary ingredients, (iii) processed foods, and (iv) ultra-processed products.

Adherence to the Mediterranean diet was assessed using the KIDMED index (Mediterranean Diet Quality Index in Children and Adolescents) [40], which is frequently used as an indicator of healthy dietary habits. This index is derived from a 16-item questionnaire that rates different dietary habits. Each response is scored according to its consistency with habits associated with the Mediterranean pattern, and the scores are summed to quantify the overall index of adherence to the Mediterranean diet (MD) of the participants. The results of the conducted test were categorized into three levels: (1) ≥8, optimal Mediterranean diet; (2) 4–7, improvement needed to adapt intakes to Mediterranean patterns; and (3) ≤3, very low diet quality.

All data were analyzed by the *Gluten3SDiet* platform and then summarized in a personalized dietary report, which was delivered to each volunteer after each data collection (at Vt0, Vt3, and Vt12). These reports provided a detailed diagnosis of their nutritional status and the quality of their diet, together with their intake of macronutrients, food groups, micronutrient deficits, and any associated risks. In addition, nutritional counseling was given through the reports with tips to achieve the dietary balance (Supplementary Figure S1).

### 2.8. Statistical Analysis

Results were statistically analyzed with the IBM SPSS statistical program, version 27 (IBM Inc., Armonk, NY, USA). The normality of the distribution was determined by the Kolmogorov–Smirnov test and homogeneity by the Levene test. Statistical analyses were conducted to calculate differences between measurements by using the Wilcoxon test in case of non-normal distribution, whereas a T-student test for paired samples was performed if data presented a normal distribution (Vt0 vs. Vt3, Vt0 vs. Vt12, and Vt3 vs. Vt12). *p* values < 0.05 were considered to be statistically significant.

## 3. Results

### 3.1. Anthropometric Parameters during the First Year of a GFD in Children and Adolescents with CD

The development of the anthropometric data of children and adolescents during their first year on a GFD is shown in Supplementary Table S1. Body weight and height values increased significantly after one year of the intervention. Even though most of the participants showed normal BMI values during the whole intervention, nearly three-quarters of participants were below P25 throughout the intervention. However, it is remarkable that the intervention decreased the number of children below P10 (72%, 60%, and 50% in Vt0, Vt3, and Vt12, respectively). All analytical measurements were under normal values at all three time points.

### 3.2. Food Frequency Consumption of Foods among Children and Adolescents with CD

Daily intakes of all food groups, with the exception of oils, were low in all subjects at Vt0 (Table 1). However, they all increased from diagnosis to Vt3 and from Vt0 to Vt12 (except for fruit, of which the intake was highest at Vt3). This increase contributed to meeting the daily intakes of cereals and dairy products, whereas intakes of vegetables and fruit remained low. Meat consumption was high at baseline. A year on a GFD with nutritional assessment reduced its intake, reaching statistical significance at Vt3 and fulfilling the recommendations. The intake of nuts was low at baseline and increased during the follow-up but did not reach the recommendation. The remaining protein sources (fish and eggs) were consumed correctly and remained unchanged after the intervention. In the case of legumes, an increase was observed after 3 and 12 months. With regard to pastries and sausages, their consumption decreased at each time point, reaching statistical significance for sausages.

### 3.3. Mediterranean Diet Quality Index According to the KIDMED Questionnaire in Children and Adolescents with CD

Participants’ adherence to the Mediterranean diet, which was calculated by the KIDMED questionnaire, showed that most participants’ diets needed improvement at baseline (Table 2). However, at Vt3 and Vt12, most participants (61.9% and 54.8% of them, respectively) had good compliance with the MD regarding the KIDMED questionnaire.

### 3.4. Food Consumption According to the NOVA System in Children and Adolescents with CD

The NOVA classification showed that all participants increased their intake of foods from group 1 and decreased their intake of foods from group 4 during follow-up (Table 3). In addition, the daily portion intake of each food group from group 4 was analyzed to obtain a deeper insight into those foods responsible for the observed reduction (Table 4). GFP had the largest contribution on all visits, followed by ultra-processed dairy products. However, this consumption decreased from Vt0 to Vt12. Furthermore, ultra-processed dairy product intake showed a trend toward lower values after the intervention. Even though participants consumed less candy and chocolate at Vt3 compared to Vt0, this change was not maintained at Vt12.

### 3.5. GFP Contribution to Total Energy Intake in Children and Adolescents with CD

According to the energy % contribution of GFP to the total calorie intake of the participants, it accounted for almost one-quarter of the total energy distribution at Vt0 (22.1 ± 8.3). This energy intake from GFP decreased after one year of dietary intervention (20.4 ± 8.1 at Vt3 and 17.7 ± 8.1 at Vt12). Statistical differences were observed from Vt0 to Vt12 and from Vt3 to Vt12 (*p* < 0.05 and *p* <0.01, respectively).

### 3.6. Energy, Macronutrient, Fiber, and Cholesterol Intakes of Children and Adolescents with CD

Table 5 presents the energy intake, macronutrient distribution, dietary fiber, and cholesterol intake of children and the percentage of those who were between the recommendations and those who were not. Energy intake was low according to each participant’s energy expenditure in 40% of participants at Vt0, 36% at Vt3, and 38% at Vt12; nevertheless, this mean energy intake increased significantly from Vt0 to Vt3 and from Vt0 to Vt12. All participants showed altered macronutrient distribution in their diets characterized by an overconsumption of protein and lipids. Even though there were significant increases in carbohydrates after the intervention of 1.3% and 0.5%, this intake did not achieve the recommendation of 50% of the total energy intake. Fiber levels increased significantly after a year on a GFD, even though most of the participants consumed the recommended amount from the beginning of the intervention. Saturated fatty acid and sugar intakes were high in most participants at each data collection.

### 3.7. Micronutrient Intakes of Children and Adolescents with CD

**Figure 1:**
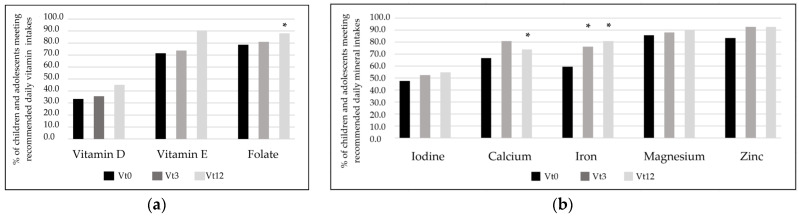
Percentage of children and adolescents with CD who accomplished more than 2/3 of the dietary reference intake of specified vitamins (**a**) and minerals (**b**). Vt0 = visit at diagnosis; Vt3 = visit after 3 months on a GFD; Vt12 = visit after 12 months on a GFD; *p* < 0.05 = Statistically significant. * above Vt3 column indicates a significant difference between Vt0 and Vt3; * above Vt12 column indicates a significant difference between Vt0 and Vt12. Percentage of children with CD and adolescents who reached or did not accomplish 2/3 of the dietary reference intakes of vitamins and minerals (suggested by the Federation of Spanish Societies of Nutrition and Dietetics (FESNAD) [37].

The results from studies in the bibliography show that children on a GFD are at greater risk of not consuming sufficient amounts of the following micronutrients: iron, vitamin D, calcium, folate, magnesium, zinc, vitamin E, and iodine [9,41]. In this sense, Figure 1 shows the consumption of the mentioned micronutrients in participants. In terms of vitamins, although participants increased the consumption of all analyzed vitamins, more than half of the participants did not fulfill the vitamin D daily recommendation. By the end of the intervention, more than 90% of participants were meeting the daily recommendation for vitamin E. Statistics showed that the increase in folate intake from 78.6% at Vt0 to 88.1% at Vt12 was significant. Among minerals, iodine intake was low at the beginning (47.6% of fulfillment), and although a slight increase was observed, it did not show a statistically significant change during the intervention. Calcium intake increased from Vt0 to Vt12. Higher intakes of iron were observed after the intervention, increasing from 59.5% to 76.2% at Vt3 and to 81% at Vt12. Throughout the intervention, more than 80% of participants met the magnesium and zinc recommendations.

## 4. Discussion

Nowadays, the available treatment for CD consists of following a GFD [42], which helps with healing the duodenal mucosa, the remission of symptoms, and the resolution of malabsorption [1]. This diet must be well-balanced and guarantee an adequate nutritional status for people with CD [43]. Nevertheless, there are some data that suggest the GFD may be an unbalanced diet both at diagnosis and after diagnosis [44,45].

With the aim of improving their dietary habits, several studies have suggested that people with CD should be given nutritional education and more continuous attention [32,46] and be assessed in long-term follow-ups [47,48]. Despite this, as far as we know, there has never been a personalized, face-to-face, and ongoing year-long dietary intervention for children newly diagnosed with CD. The current dietary intervention and follow-up implemented positive dietary changes in the intake of certain food groups, reduced the consumption of UPF, and increased adherence to the MD after one year.

The consumption of fruit, vegetables, dairy products, and cereals increased in the present intervention, reaching correct daily intakes in the case of the last two food groups. Legume and nut intake increased, which could indicate that the intervention promoted it, as they are a good source of proteins and micronutrients [49]. These data are in part similar to those found in other studies, where the dietary habits of children with CD had been analyzed at one point in time. Our previous studies showed that the consumption of cereals, fruit, and vegetables in children with CD was low and underlined that most participants did not meet the recommendations. Animal-based foods, especially meat, were consumed in excess [22,31]. In the same line, Lionetti et al. observed that children with CD did not achieve the minimum food portions recommended in their country according to pulses, vegetables, eggs, and fish, while those of sugary drinks, meat, and processed meat were exceeded. Moreover, it should be noted that when the celiac population´s diet has been compared to that of healthy controls, it has been less balanced [46].

These dietary changes were in accordance with data from NOVA analysis. Participants consumed more fresh and unprocessed foods belonging to NOVA classification group 1 after the intervention, which is in line with other studies [50]. In the present study, for example, whole grains such as maize or buckwheat flour, which are naturally gluten-free cereals or pseudo-cereals from NOVA group 1, were used by several participants in their homemade bread. One of the recommendations of the interventionists was always to encourage them to prepare their food at home, especially cereal derivatives, with as few processed ingredients as possible. This is in line with the suggestion by Di Nardo et al. [9] that nutritional counseling should promote the use of local, naturally GF foods to support a diet that is not only more balanced but also economically viable.

On the other hand, UPF consumption, from the NOVA4 group, was reduced by the end of the intervention, which is in line with another interventional study [50]. The literature suggests [51,52,53] that dietary changes resulting from the introduction of UPF not only lead to the development of several pathologies such as cardiovascular diseases, diabetes, and cancer but also replace the intake of fresh foods that form the basis of traditional diets [54,55]. Therefore, it is highly recommended to avoid its consumption not only in the population with CD but also in the general population [56,57,58,59].

To gain deeper insights into which UPFs were the most consumed, an analysis of foods from NOVA group 4 was carried out. As a result, GFPs contributed the most as sources of UPF. Among them, biscuits and pastries, which were the most consumed foods at the beginning, were reduced after the intervention. Taking into account that cereal intake increased, it can be suggested that participants consumed more bread, pasta, or other carbohydrate sources such as rice or other naturally non-gluten-containing cereals, but not biscuits. The literature suggests that the consumption of biscuits by children with CD represents a high percentage of total energy intake and that GFP ingestion contributes between 24 and 36% of total daily calorie intake [22,46]. Another study found that the most common GFPs consumed by Spanish children and adolescents were bread and fine bakery ware products [60]. As far as we know, there are no longitudinal and interventional studies in the literature analyzing the consumption of different GFPs in children. The results from the present study highlight the relevance of managing GFP consumption from the time of diagnosis and providing nutritional education on these foodstuffs.

These dietary data were supported by the results from the KIDMED questionnaire. The number of participants that fully adhered to the MD increased after the intervention increased, being higher than 50% of the sample size, thus indicating the effectiveness of the ongoing nutritional assessment.

As expected, changes in food group consumption also affected the energy intake and nutrient profile of participants´ diets. The present research shows that, in general, participants’ energy intake did not match their energy expenditure, although it was enhanced during the intervention. Studies in the literature have found controversial data concerning energy intake. Zuccotti et al. [61] found that the average energy intake was higher in people with CD compared with healthy people. In contrast, other studies [62,63] described a reduction in daily energy intake in people with CD.

In relation to the nutrient intake, increased consumption of all plant-based foods, especially that of non-processed cereals, was related to a higher carbohydrate and fiber intake at the end of the intervention, although this remained below 50% of the total energy intake. Our findings on low carbohydrate intake were similar to those of other researchers [64,65,66]. The low intake of carbohydrates could be explained by the need to avoid several gluten-containing foods, for instance, generic bread or pasta made wth gluten-containing flours, and their choice of alternative GFPs.

The existing literature on dietary fiber ingestion in children with CD may be contradictory. Several studies [45,67] have indicated the proper intake of fiber, whereas others [68] claimed a low intake of it because people with CD do not consume enough fruits and vegetables, as we observed. So, these findings together with the trends [69] suggest that by providing nutritional education to children with CD, the consumption of this type of fresh food source of fiber can rise.

Excessive meat consumption could be responsible for the high intake of protein, fats, and saturated fats. Pastries and the observed increase in dairy products (which were mainly whole milk) could also contribute as a source of saturated fats. A case-control study found that protein-rich foods such as meat, fish, and eggs were consumed in greater quantities by children with CD than by healthy controls [70]. Regarding studies where the lipid profile has been analyzed, Forchielli et al. suggested that most saturated fats came from dairy products in the diets of children with CD [67]. In relation to unsaturated fats, the increase in nut consumption could lead to a higher fulfillment of these nutrients, especially polyunsaturated ones.

The literature suggests that children are at risk of inadequate iron, vitamin D, folate, and calcium intake and that these inaccuracies may be worsened by a GFD [9,26]. According to another study, children with CD showed very low intakes of iodine, vitamin E, vitamin D, and folate [41], suggesting the need for more significant involvement of a dietitian in the management of CD. The low intake of vitamin D observed in the present study is in accordance with the current literature [63,70,71]. However, the intervention promoted an increase in milk and dairy, which resulted in a better accomplishment of vitamin D, together with that of calcium. Insufficient dietary folate has been observed to also be common in the young population with CD [72]. However, in the current study, since the intake of green leafy vegetables and whole grains (non-processed cereals) increased, dietary folate levels improved. In the case of iron, the increase in legumes could have increased this micronutrient recommendation. Vitamin E, which has been observed to be low in the pediatric population with CD studied at one point in time [25], achieved correct amounts in the present study, which could be linked to the increase in both olive oil and nut ingestion. Iodine intake is known to be deficient in the general population [73], and although a slight increase may have been due to the improved consumption of dairy products and nuts, it was insufficient and similar to the results of other studies [71]. Delvecchio et al. concluded that the need for adequate iodine ingestion in people with CD should be regularly emphasized to minimize the risk of its deficiency [74]. For magnesium and zinc, in contrast to other studies that found low intakes of these micronutrients [68,75], our participants met the recommendations, and the observed increase during the intervention could be due to the higher consumption of whole grains, nuts, or legumes. Taken as a whole, the present study demonstrates that ongoing and face-to-face follow-ups could help in the achievement of micronutrient recommendations.

This study presents some limitations. First, the selection of intense physical activity for all participants in the calculation of total energy expenditure could be the reason for some participants not fulfilling energy requirements. Secondly, another difficulty of working with humans is that dietary recall mistakes, including errors in estimating portions consumed and omitting ingredients or foods (either intentionally or unintentionally), could lead to the underestimation of nutrient intakes, as with any food recall. Moreover, a control group of children with celiac disease who did not receive any intervention is lacking. This would be an interesting inclusion to observe the effectiveness of the intervention. Nevertheless, comparing data after the intervention (Vt12) to those without the intervention (Vt0, at diagnosis) could also be useful to measure the effectiveness of the intervention. Lastly, it has to be noted that another limitation of this work is that the selection of the people who took part in this research was not randomized as they were volunteers. This fact could affect the recorded dietary habits of the participants, as people who participated tended to be more interested in nutrition than the rest of the population. However, it is important to emphasize that this study offers valuable information on areas that dietitians should work on, particularly in the area of plant-based food consumption, which should be targeted by future interventions.

## 5. Conclusions

Taking all of the above factors into account, it has been observed that a correct personalized nutritional assessment, nutritional education, and continuous follow-up of children with CD by dietitians are crucial factors in achieving positive results in dietary balance. In the current intervention, the results were positive, as the intake of fresh foods increased after a year of the intervention and the intake of the less nutritious food groups, such as UPF, decreased. These changes may indicate that the intervention was effective at changing most dietary patterns.

## Figures and Tables

**Table 1:** Food group consumption frequency of children and adolescents with CD (mean of portions/day or week ± SD).

		Children and Adolescents				*p* Value		
		Recommended Daily Portion Intake *	Vt0	Vt3	Vt12	Vt0 vs. Vt3	Vt0 vs. Vt12	Vt3 vs. Vt12
**Daily consumption**	**Dairy**	2–4	1.9 ± 0.4	2.3 ± 0.6	2.4 ± 0.5	<0.001	<0.001	NS
	BRI		36%	14%	26%			
	RI		64%	86%	74%			
	ARI		0%	0%	0%			
	**Cereals**	4–6	3.3 ± 0.8	4 ± 0.8	4 ± 1.9	<0.001	0.001	NS
	BRI		60%	26%	33%			
	RI		40%	74%	67%			
	ARI		0%	0%	0%			
	**Vegetables**	2	0.6 ± 0.4	0.9 ± 0.2	1 ± 0.5	<0.001	<0.001	NS
	BRI		95%	86%	81%			
	RI		5%	14%	19%			
	ARI		0%	0%	0%			
	**Fruits**	3	1.7 ± 0.6	2.1 ± 0.6	1.8 ± 0.6	<0.05	<0.05	NS
	BRI		90%	83%	83%			
	RI		10%	17%	17%			
	ARI		0%	0%	0%			
	**Oils**	3–6	3 ± 0.5	3.3 ± 0.7	3.4 ± 0.6	<0.05	<0.05	NS
	BRI		26%	10%	5%			
	RI		74%	90%	95%			
	ARI		0%	0%	0%			
**Weekly consumption**	**Meat**	3–4	4.4 ± 1.8	4 ± 1.8	4.2 ± 1.3	<0.05	NS	NS
	BRI		2%	10%	2%			
	RI		45%	57%	45%			
	ARI		53%	33%	53%			
	**Fish**	3–4	3 ± 2	3.1 ± 1.7	3.1 ± 1.9	NS	NS	NS
	BRI		41%	36%	40%			
	RI		38%	48%	46%			
	ARI		21%	16%	14%			
	**Eggs**	3–4	3.1 ± 0.5	3 ± 0.4	3 ± 0.4	NS	NS	NS
	BRI		10%	12%	12%			
	RI		71%	81%	69%			
	ARI		19%	7%	19%			
	**Legumes**	2–4	2.2 ± 1.2	2.4 ± 1.1	2.7 ± 1	<0.05	<0.05	<0.05
	BRI		33%	24%	19%			
	RI		67%	76%	74%			
	ARI		0%	0%	7%			
	**Nuts**	3–7	1.5 ± 2.1	2.6 ± 2.1	2.4 ± 2.3	<0.001	<0.05	NS
	BRI		67%	36%	50%			
	RI		33%	62%	48%			
	ARI		0%	2%	2%			
	**Pastries**		4.7 ± 3	4.1 ± 3.5	3.9 ± 3.9	NS	NS	NS
	**Sausages**		3.3 ± 2	2.1 ± 1.7	2.5 ± 1.2	<0.05	0.056	NS

Abbreviations: *p* < 0.05 = Statistically significant; NS = Not Significant. BRI: below recommended intakes: RI: Recommended intakes fulfilled; ARI: above recommended intakes. Vt0 = visit at diagnosis; Vt3 = visit after 3 months on a GFD; Vt12 = visit after 12 months on a GFD. * Recommended intake according to the Spanish Society of Community Nutrition (SENC) [38].

**Table 2:** Mediterranean Diet Quality Index statistics for the total sample of children and adolescents with CD.

		Children and Adolescents			*p* Value	
		Vt0	Vt3	Vt12	Vt0 vs. Vt3	Vt0 vs. Vt12
**Total punctuation**		6.5 ± 2.1	7.9 ± 1.4	7.7 ± 2.1	<0.001	<0.001
**KIDMED** **index score**	Poor (≤3)	10%	0%	2%		
	Average (4–7)	55%	38%	43%		
	Good (≥8)	35%	62%	55%		

Abbreviations: Vt0 = visit at diagnosis; Vt3 = visit after 3 months on a GFD; Vt12 = visit after 12 months on a GFD; *p* < 0.05 = Statistically significant.

**Table 3:** Food consumption according to NOVA classification in children and adolescents with CD (mean of portions/day ± SD).

	Children and Adolescents			*p* Value		
	Vt0	Vt3	Vt12	Vt0 vs. Vt3	Vt0 vs. Vt12	Vt3 vs. Vt12
**G1**	6.6 ± 2	7.2 ± 2	7.1 ± 1.9	<0.05	<0.05	NS
**G2**	2.7 ± 0.7	2.9 ± 1.1	2.7 ± 0.9	NS	NS	NS
**G3**	0.7 ± 0.4	0.7 ± 0.5	0.9 ± 0.7	NS	<0.05	<0.05
**G4**	4.5 ± 1.3	4 ± 1.5	3.8 ± 1.5	<0.05	<0.05	NS

Abbreviations: Vt0 = visit at diagnosis; Vt3 = visit after 3 months on a GFD; Vt12 = visit after 12 months on a GFD; G1: Group 1 (unprocessed or minimally processed foods); G2: Group 2 (processed culinary ingredients); G3: Group 3 (processed foods); G4: Group 4 (ultra-processed products). *p* < 0.05 = Statistically significant; NS = Not Significant.

**Table 4:** Daily portion consumption of each ultra-processed food group (G4) among children and adolescents with CD.

	Children and Adolescents			*p* Value		
	Vt0	Vt3	Vt12	Vt0 vs. Vt3	Vt0 vs. Vt12	Vt3 vs. Vt12
**GFP**	2.5 ± 0.9	2.3 ± 0.9	2.3 ± 0.9	NS	<0.05	NS
**Ultraprocessed dairy products**	0.8 ± 0.6	0.6 ± 0.6	0.5 ± 0.5	NS	0.06	NS
**Snacks**	0.1 ± 0.2	0.1 ± 0.2	0.2 ± 0.2	NS	NS	NS
**Candy and chocolates**	0.3 ± 0.4	0.2 ± 0.3	0.3 ± 0.4	<0.05	NS	NS
**Sausages**	0.6 ± 0.5	0.4 ± 0.4	0.5 ± 0.4	NS	NS	NS
**Other ultraprocesed foods (soft drinks, juices, ultra processed foods and sauces)**	0.2 ± 0.2	0.1 ± 0.2	0.1 ± 0.2	NS	NS	NS

Abbreviations: GFP: Gluten-free product; Vt0 = visit at diagnosis; Vt3 = visit after 3 months on a GFD; Vt12 = visit after 12 months on a GFD; NS = Not Significant.

**Table 5:** Energy, macronutrient, and micronutrient daily intake of children and adolescents with CD (mean ± SD).

		Children and Adolescents			*p* Value		
	Recommended Intake *	Vt0	Vt3	Vt12	Vt0 vs. Vt3	Vt0 vs. Vt12	Vt3 vs. Vt12
Energy intake (kcal)	±20% of EE	1508.4 ± 175.1	1678.1 ± 253	1710.1 15 ± 207.9	<0.001	<0.001	NS
<80%		40%	36%	38%			
80–120%		48%	50%	45%			
>120%		12%	14%	17%			
Protein (%)	10–15	16.2 ± 2.2	15.7 ± 2	16.7 ± 2.1	NS	NS	NS
<10%		0%	0%	0%			
10–15%		24%	43%	26%			
>15%		76%	57%	74%			
Lipids (%)	30–35	38 ± 3.9	37.3 ± 5.2	37.1 ± 4.4	NS	NS	NS
<30%		7%	9%	5%			
30–35%		17%	29%	24%			
>35%		76%	62%	71%			
Saturated fats (%)	<10	11.4 ± 2.1	10.5 ± 2.6	11.4 ± 2.4	NS	NS	NS
<10%		33%	48%	36%			
≥10%		67%	52%	64%			
Monounsaturated fats (%)	≥20	15.1 ± 2.6	14.5 ± 3.6	15.1 ± 3.4	NS	NS	NS
<20%		90.5%	83%	90%			
≥20%		9.5%	17%	10%			
Polyunsaturated fats (%)	≥4	4.1 ± 0.8	4.2 ± 1.5	4.2 ± 1	NS	NS	NS
<4%		45%	55%	38%			
≥4%		55%	45%	62%			
Carbohydrates (%)	50–60	45.7 ± 4.1	47 ± 5.6	46.2 ± 4.5	<0.05	NS	NS
<50%		83%	67%	74%			
50–60%		17%	33%	26%			
>60%		0%	0%	0%			
Simple sugars (%)	<10	12.5 ± 3.4	11.8 ± 4.4	12.9 ± 3.7	NS	NS	<0.05
<10%		29%	29%	21%			
≥10%		71%	71%	79%			
Fibre (g)	g/day	18.1 ± 6.4	20.5 ± 8.3	21.9 ± 6.7	NS	<0.05	NS
<10 g/1000 kcal		29%	26%	21%			
10–14 g/1000 kcal		52%	57%	48%			
>14 g/1000 kcal		19%	17%	31%			
Cholesterol (mg)	<300	240 ± 93.3	280 ± 83.2	272.89 ± 80.2	NS	<0.05	NS
<300		36%	55%	52%			
≥300		64%	45%	48%			

Abbreviations: Vt0 = visit at diagnosis; Vt3 = visit after 3 months on a GFD; Vt12 = visit after 12 months on a GFD; EE = Energy Expenditure. *p* < 0.05 = Statistically significant; NS = Not Significant. * Recommended contribution to a balanced diet proposed by the Federation of Spanish Societies of Nutrition and Dietetics (FESNAD) [37] and Spanish Society for Community Nutrition (SENC) [38].

## Data Availability

Data are contained within the article and Supplementary Materials.

## Supplementary Materials

The following supporting information can be downloaded at: https://www.mdpi.com/article/10.3390/nu16152418/s1, Supplementary Table S1: Anthropometric measurements in children and adolescents with CD. Supplementary Figure S1: Image of a personalized dietary report.
