# Supplementary material for: Importance of an Ongoing Nutritional Counselling Intervention on Eating Habits of Newly Diagnosed Children with Celiac Disease
Source: Nutrients. 2024 Jul 25;16(15):2418. doi: 10.3390/nu16152418 (PMC11314293; doi:10.3390/nu16152418)

Supplementary Table S1. Anthropometric measurements in children and adolescents with CD.

		**Children and adolescents**			**p value**	
**Anthropometric measurements**	**Reference values ***	**Vt0**	**Vt3**	**Vt12**	**Vt0 vs. Vt3**	**Vt0 vs. Vt12**
Weight (kg)		26.1 ± 10.4	27.6 ± 10.8	30.3 ± 11.2	NS	<0.001
	≤ P3	33%	23%	12%		
	P10	14%	16%	25%		
	P25	23%	18%	21%		
	P50	19%	22%	32%		
	P75	9%	9%	5%		
	≥P85	2%	2%	5%		
Height (cm)		125.1 ± 19	127.4 ± 19.2	132.1 ± 18.8	NS	<0.001
	≤ P3	19%	30%	14%		
	P10	24%	9%	23%		
	P25	19%	5%	9%		
	P50	14%	37%	26%		
	P75	12%	5%	12%		
	≥P85	12%	2%	16%		
BMI (kg/m^2)		16 ± 2.2	16.3 ± 2.2	16.7 ± 2.1	<0.05	<0.05
	≤ P3	3%	0%	0%		
	P10	21%	19%	7%		
	P25	48%	41%	43%		
	P50	19%	31%	40%		
	P75	7%	7%	5%		
	≥P85	2%	2%	5%		

Abbreviations: Abbreviations: Vt0 = visit at time 0, at diagnosis; Vt3 = visit after 3 months on a GFD; Vt12 = visit after 12 months on a GFD; $p < 0.05$ = Statistically significant; NS = Not Significant; BMI = Body Mass Index. * According to Sobradillo et al., 2004.

Supplementary Figure S1. Image of a dietary report of a participant.

Participant and code: XX

Visit time and date: XXX

UPV EHU

Osakidetza

Asociación de Celíacos y Sensibles al Gluten

ANTHROPOMETRY

Weight (kg): X

Height (cm): X

BMI (kg/m²): X

BMI: Body Mass Index

BMI percentile: Underweight | Normal weight | Overweight | Obesity (< p3 — p50 — p85 — p95 <)

Fat mass (%): X

Fat mass percentile: Low | Normal | Overweight | Obesity (p10-p25 — p75-p85 — p90 <)

Comments:

ENERGY AND NUTRIENT CONSUMPTION

- **Daily energy intake (kcal):** 2000

According to our calculations, your daily energy intake (the calories we take in during the day) was around 2000 kcal; this would be sufficient for your daily energy expenditure (the calories we spend during the day), which should be around 1800-2200 kcal per day.

- **Energy distribution: (1) Macronutrients**

The energy you have consumed, around XX kcal, comes from different foods and these foods are made up of different nutrients. In the case of macronutrients, your consumption was as follows:

	Recommended % and foods containing them	Your consumption
Carbohydrates	%50-60: Naturally gluten-free cereals, tubers, pulses, fruit and vegetables	% 50
Proteins	%10-15: Animal-based foods	% 11
Fats	%30-35: Olive oil, nuts, avocado	% 36

Your energy distribution is within the recommended range. You eat foods rich in carbohydrates, such as bread, potatoes and fruit. It should also be noted that most of the food you consume is of healthy origin, i.e. you consume mostly fresh food and very little processed food.

- **(2) Micronutrients:** They are vitamins and minerals that the body needs in very small amounts. A deficiency in any of them can cause serious and even life-threatening conditions. We have noticed that you are missing some of them (calcium), and we would like to encourage you to get them by eating different food groups, which will be highlighted in the right part of the form.

- **Fiber** (g): (10-14 g/1000kcal): **Adequate**

Your fiber intake meets the recommendations. You eat two portions of fruit and one portion of vegetables a day, both of which are sources of fiber.

FOOD GROUP (FG) CONSUMPTION

- FG to eat **every day** and at **every main meal:**

FG	Recommended intake	Your consumption
Cereals	4-6 portions/day	3
Vegetables	2 portions/day	2
Fruits	3 portions/day	3
Oils (olive oil consumption as priority)	3-6 portions/day	>3

- FG to eat **every day** and/or **weekly**:

Dairy products	2-4 portions/day	1
Meat	3-4 portions/week of each FG (alternating its consumption)	3-5
Fish		3-4
Eggs		3-4
Legumes	2-4 portions/week	2-4
Nuts	3-7 portions/week	Do not eat

- FG to eat **occasionally:**

Processed meats/sausages	Almost daily
Pastries, bakery products	Ocasionally
Other ultra-processed foods	Ocasionally

Inadequate consumption | Almost adequate consumption | Adequate consumption

Recommended intakes according to the Spanish Society of Nutrition (SENC). 2011 Rev Esp Nutr Comunitaria 2011;17(4):178-199.

RECOMMENDATIONS FOR A HEALTHY DIET

FG	What to do?	Our recommendation
**Cereals**	Increase naturally gluten-free (GF) cereals	Even if you eat foods from the cereal group,we recommend that you increase your consumption of naturally GF cereals whenever possible. You can add a grain such as quinoa or rice to salads, make buckwheat pancakes, etc.
**Vegetables**	Include raw vegetables in your diet	The consumption of vegetables should be daily and you are following the recommendations, how good! Remember that the ideal is to eat one portion raw and one cooked every day, so keep it up!
**Fruits**	Good intake!	We have seen that you have included three pieces of fruit a day in your diet, we can only advise you to continue with this as most children do not normally eat three pieces of fruit a day. We would like to point out that you are also including different types of fruit, including citrus fruits.
**Oils**	Good intake!	Olive oil is a super healthy source of fat and we should eat it, preferably raw. Continue to use it at home.
**Dairy products**	Increase its intake	You eat one portion of dairy products a day, but we recommend that you add at least one more portion. This will keep your calcium levels slightly higher. Calcium is very important for maintaining the correct structure, especially of teeth and bones, and it has been seen that these levels can be lower in people with coeliac condition.
**Meat**	Good intake!	You have a good intake of meat. Do not eat more than one portion of red meat per week.
**Fish**	Good intake!	You have a good intake of fish.
**Eggs**	Good intake!	You have a good intake of eggs.
**Legumes**	Good intake!	You tell us that you eat pulses 2-4 times a week. It is recommended that the dessert of each meal is a fruit rich in vitamin C (citrus fruits) to improve the absorption of iron.
**Nuts**	Start including them in the diet!	We encourage you to include nuts in your diet. You can eat them as a snack, in yoghurt or as a spread to fill sandwiches, thus reducing the consumption of cold meats.
**FG to eat occasionally**	Reduce sausage consumption	Try to reduce your sausage consumption.

SUMMARY: In short, your diet is quite varied. We encourage you to eat more cereals, to add one or two portions of dairy products a day and to start eating nuts. However, we would advise you to reduce your intake of cold meats and fill your sandwiches with other foods: nut creams, vegetables, eggs, tuna, avocado...

DIETARY RECOMMENDATIONS

Food group	Recommended intake	Weight of each portion(raw and net)	Home-based measure (a intake)
**CEREALS** (bread, rice, pasta, cereals and flour, potatoes...) 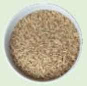	4-6 portions/day<br><br>*Give priority to gluten-free whole grains 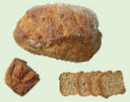	40-60 g. bread<br>60-80g of rice<br>60-80g of pasta (macaroni, spaghetti...)<br>30g breakfast cereals<br>150-200g potatoes<br><br>*Give priority to whole grains	3-4 slices<br>1 normal cooked dish<br>1 plain cooked dish<br>2-3 tablespoons<br>1 large or 2 small potatoes 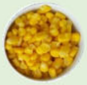
**VEGETABLES** 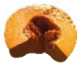	2 portions/day 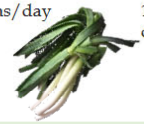	150-200 g of raw or cooked vegetables 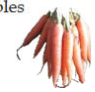	1 plate of mixed salad<br>1 plate of cooked vegetables<br>1 large tomato<br>2 carrots
**FRUITS** 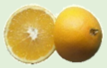 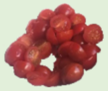	3 portions/day 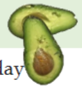	100-200 g<br><br>*If possible, eat the fruit without peeling.	1 medium apple or orange<br>1 cup cherries, strawberries<br>2 slices of melon
**OILS**	3-6 portions/day	10 ml 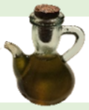	1 tablespoon
**DAIRY PRODUCTS** 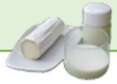	2-4 portions/day	200ml milk<br>125g yoghurt<br>80-125g fresh cheese<br>40-60g of cured cheese	1 glass<br>2 units<br>1 single serving<br>2-3 slices of cheese

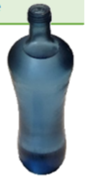

As for **water**, it is advisable to have a full bottle at hand to stay hydrated. Always prioritize its consumption over juices or soft drinks.

Food group	Recommended intake	Weight of each portion (raw and net)	Home-based measure (a intake)
**MEAT** 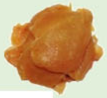		100 - 125 g	1 small fillet<br>1 quarter chicken<br>1 quarter rabbit
**FISH** 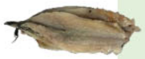	3-4 portions/ week of each FG (alternating its consumption)	125- 150 g 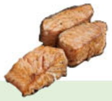	1 small fillet
**EGGS**		A medium-size egg (53-63 g.)	1 unit 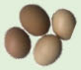
**LEGUMES** 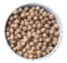	2-4 portions/week 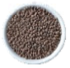	60 - 80 g raw beans, chick peas, lentils	1 normal cooked dish
**NUTS**	3-7 portions/week	20 - 30 g. 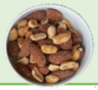	1 handful or single serving

Occasional consumption of sausages and fatty meats; sweets, snacks, soft drinks and sugary drinks; margarine and pastries. Remember, the fewer ultra-processed foods, the better!

It is advisable to avoid **cooking techniques** that use a lot of fat, such as frying, breading, battering, etc. Use olive oil whenever possible. It is preferable to use techniques such as steaming, boiling, grilling or baking that do not require a lot of oil.

¡ **Remember to stay active!** Try to move around a lot and spend less time sitting in front of the TV.

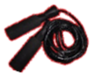 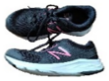

Supplement: Supplementary file 1 [file nutrients-16-02418-s001.zip › nutrients-3101140-supplementary.pdf]
